# Insights into the Role of Glutathione Peroxidase 3 in Non-Neoplastic Diseases

**DOI:** 10.3390/biom14060689

**Published:** 2024-06-13

**Authors:** Nan Zhang, Haihan Liao, Zheng Lin, Qizhu Tang

**Affiliations:** 1 Department of Cardiology, Renmin Hospital of Wuhan University, Wuhan 430060, China; 2011302180404@whu.edu.cn (N.Z.); liaohaihan@whu.edu.cn (H.L.);; 2 Hubei Key Laboratory of Metabolic and Chronic Diseases, Wuhan 430060, China

**Keywords:** glutathione peroxidase 3, oxidative stress, reactive oxygen species, gene expression, non-neoplastic disease

## Abstract

Reactive oxygen species (ROSs) are byproducts of normal cellular metabolism and play pivotal roles in various physiological processes. Disruptions in the balance between ROS levels and the body’s antioxidant defenses can lead to the development of numerous diseases. Glutathione peroxidase 3 (GPX3), a key component of the body’s antioxidant system, is an oxidoreductase enzyme. GPX3 mitigates oxidative damage by catalyzing the conversion of hydrogen peroxide into water. Beyond its antioxidant function, GPX3 is vital in regulating metabolism, modulating cell growth, inducing apoptosis and facilitating signal transduction. It also serves as a significant tumor suppressor in various cancers. Recent studies have revealed aberrant expression of GPX3 in several non-neoplastic diseases, associating it with multiple pathological processes. This review synthesizes the current understanding of GPX3 expression and regulation, highlighting its extensive roles in noncancerous diseases. Additionally, this paper evaluates the potential of GPX3 as a diagnostic biomarker and explores emerging therapeutic strategies targeting this enzyme, offering potential avenues for future clinical treatment of non-neoplastic conditions.

## 1. Introduction

Reactive oxygen species (ROSs) are the products of the one-electron reduction of molecular oxygen, generated by several oxidase enzymes and the mitochondrial respiratory chain. These include superoxide anion (O_2_^−^) and hydrogen peroxide (H_2_O_2_) [1]. ROSs play dual roles within biological systems; they act as essential signaling molecules regulating vital physiological processes such as cell proliferation, differentiation and apoptosis [2], However, at pathologically high levels, ROSs can contribute to the development of diseases such as cancer, pulmonary disease and cardiac disorders [2,3,4]. Therefore, maintaining an optimal balance of ROSs is crucial for cellular health.

The cellular damage inflicted by ROSs is influenced not only by their intracellular concentration but also by the balance between ROSs and endogenous antioxidant mechanisms. Antioxidants form the primary defense against ROS-induced cellular damage [5]. The body’s antioxidant defense system consists of both enzymatic and non-enzymatic components. Enzymatic antioxidants mainly include superoxide dismutases (SODs), catalase and glutathione peroxidases (GPXs) [6,7]. SODs play a key protective role in cellular defense mechanisms by catalyzing the dismutation of O_2_^−^ into H_2_O_2_ and molecular oxygen. While H_2_O_2_ is a relatively mild oxidant, its enzymatic or non-enzymatic conversion can generate more reactive ROSs, such as hypochlorous acid and hydroxyl radicals, These highly reactive ROSs have the potential to cause substantial cellular damage, including DNA degradation and apoptosis [8]. However, through the action of GPXs and catalase, H_2_O_2_ is efficiently converted into water, preventing its transformation into more deleterious ROSs [9]. Consequently, this GPX-mediated conversion process underscores the criticality of maintaining a delicate balance between ROS production and antioxidant defense mechanisms in cellular homeostasis.

## 2. GPXs

GPXs, discovered in 1957, comprise an enzyme family with eight members (GPX1-GPX8) characterized by similar sequences, biochemical functions and a catalytic domain consisting of selenocysteine/cysteine, glutamine and tryptophan [10,11]. These enzymes exhibit genetic, structural and functional diversity, fulfilling both common and distinct roles within the body [10]. Phylogenetic analysis categorizes GPXs into three evolutionary groups, GPX1/GPX2, GPX3/GPX5/GPX6 and GPX4/GPX7/GPX8, with only GPX1-4 and GPX6 identified as selenoproteins [10,12].

GPXs exert antioxidant effects in different locations in the body and in different cell compartments (Table 1 summarizes the different types of GPX, their peroxidatic residues, gene locations, tissue distributions, cellular localizations and corresponding references). GPX1, one of the most abundant members of the GPX family, is widely expressed in almost all cells and is distributed in the cytoplasm, mitochondria and peroxisomes [13]. GPX2 is primarily localized in the intestinal and pulmonary epithelium, with notable expression at the bases of intestinal crypts [14]. Approximately 70% of GPX3, the predominant form in the basolateral compartment of kidney cells [15], is secreted by the basolateral membrane of proximal convoluted tubule cells [16]. GPX4 is present in the cytosol, mitochondria and nucleus of cells in the testes, spermatozoa and brain [17]. GPX5 is secreted in the epididymis and spermatozoa, while GPX6 is localized in the olfactory epithelium [18,19]. GPX7 and GPX8, both localized in the endoplasmic reticulum, share many characteristics: both contain cysteine instead of selenocysteine in their catalytic centers and exhibit minimal GPX activity due to the absence of a GSH-binding domain [20,21]. Many cysteine-based GPX-homologous sequences have been discovered, which do not rely on GSH as a reductant but prefer redoxins characterized by a CxxC motif [22].

GPX family members have long been a research focus in the field of oxidative stress. GPX3, a key member of the GPX family, has predominantly been investigated within oncology [23,24,25], but it has also been shown to play essential roles in pathological processes such as inflammation, fibrosis, metabolic homeostasis and insulin resistance [26,27,28]. This review focuses on the expression, regulation and evolution of GPX3 in non-neoplastic diseases, assesses its potential as a prognostic and diagnostic biomarker and explores new therapeutic avenues targeting GPX3.

## 3. Basic Information on GPX3

### 3.1. Discovery and Nomenclature of GPX3

**Figure 1:**
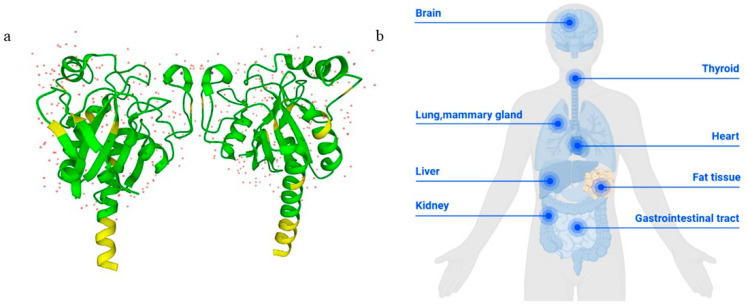
Structure and tissue distribution of the GPX3 protein. (**a**) The crystal structure of GPX3 (2R37) from the Protein Data Bank. (**b**) GPX3 is widely distributed in the kidney, retina, lungs, liver, gastrointestinal tract, heart, brain, thyroid, mammary gland and adipose tissue.

GPX3, also known as plasma glutathione peroxidase (GPX-P) or extracellular glutathione peroxidase (eGPX), is a unique member of the GPX family found extracellularly, notably in plasma [29]. The human GPX3 protein is a homotetramer composed of 226 amino acids, with a molecular weight of 21.5 kDa, distinguishing it as the only secreted enzyme among its peers [30] (Figure 1a).

### 3.2. Expression and Distribution of GPX3 in Cells

GPX3 is a selenoprotein antioxidant enzyme synthesized primarily in the kidneys, from where it is transported into the systemic circulation. Its expression extends beyond plasma, permeating various tissues, including the kidney, retina, thyroid, lungs, adipose tissue, heart, liver, gastrointestinal tract, brain, mammary gland and appendix [31] (Figure 1b). GPX3 is not only secreted into extracellular fluids such as breast milk, amniotic fluid, atrial fluid and follicular fluid of the thyroid gland but also binds specifically to the basement membrane of certain tissues such as the gastrointestinal tract, lung and male genital tract [32]. Interestingly, in the lungs, GPX3 selectively binds only to the basement membrane of type II pneumocytes, not type I pneumocytes, indicating tissue-specific interactions, although the structural basis for this specificity remains unclear [32].

### 3.3. Regulation of GPX3 Expression

**Figure 2:**
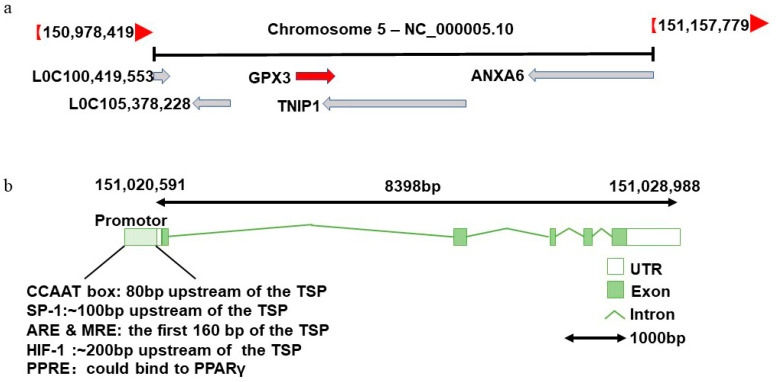
The structure of the GPX3 gene. (**a**) GPX3 is localized on chromosome 5q33.1. The red arrow marks the transcriptional direction of GPX3. (**b**) Gene structure of GPX3. The GPX3 gene is 8398bp long and contains five exons. The promoter region contains the CCAAT box, and binding sites of SP-1, ARE, MRE, HIF-1 and PPRE. TSP: transcription start site of the promoter; ARE: antioxidant response element; MRE: metal response element; HIF-1: hypoxia-inducible factor-1; PPRE: peroxisome proliferator response element; PPARγ: proliferator-activated receptor γ.

The human *GPX3* gene spans approximately 8.4 kb, comprises five exons and is located on chromosome 5q33.1 [33,34] (Figure 2a). Early studies identified a transcription start site 298 bp upstream of the start codon [34]. Subsequent research by Bierl et al. revealed an additional transcription start site 233 bp downstream of this promoter, with a 25-fold increase in transcriptional activity [35]. Notably, this new promoter region contains a classical CCAAT box 80 bp upstream and a GC-enriched region near the Sp-1 binding site approximately 100 bp upstream of the transcription start site. Additionally, the promoter features an antioxidant response element (ARE) and metal response element (MRE) within the first 160 bp of the transcription start point, which respond to oxidative stimuli such as H_2_O_2_ and tert-butylhydroquinone [35]. ARE has been identified as a DNA-binding site for nuclear factor E2-related factor 2 (Nrf2), an antioxidant transcription factor that mediates ARE-driven upregulation of antioxidant genes and may act as a major regulator of the antioxidant cellular stress response [36]. Although Nrf2 binds to the ARE, GPX3 is not a target of Nrf2 [37]. Moreover, a hypoxia-inducible factor-1 (HIF-1) binding site was found approximately 200 bp upstream of the transcription start site, suggesting that hypoxia is a strong transcriptional regulator of GPX3 expression [35]. Compared to normal-oxygen conditions, GPX3 expression levels under hypoxic conditions increased nearly threefold after 24 h [38]. The peroxisome proliferator response element (PPRE) was also identified in the human GPX3 promoter region, indicating that peroxisome proliferator-activated receptor γ (PPARγ) can directly regulate GPX3 expression by binding to the PPRE [39] (Figure 2b).

GPX3 expression is also regulated by epigenetics. It was found in multiple tumor cell lines that hypermethylation of the GPX3 promoter CpG island resulted in downregulation or complete silencing of GPX3 expression [40]. Additionally, various miRNAs are involved in the posttranscriptional regulation of GPX3. MiR-483-5p binds to nucleotides 570-576 of the 3′ UTR of GPX3 to inhibit GPX3 protein synthesis [41]. In addition, both miR-196a and miR-921 can target the GPX3 3′-UTR to regulate protein expression [42,43]. In conclusion, the regulation of GPX3 expression can be influenced by multiple factors, and the regulatory mechanisms are vary in different cells.

### 3.4. GPX3 Function

GPX3 is a selenoprotein with antioxidant activity that detoxifies hydrogen peroxide and organic hydroperoxides, reducing oxidative stress by converting these compounds into less reactive molecules. This selenoprotein plays a critical role in mitigating oxidative damage in extracellular spaces, thus protecting cellular components from oxidative stress.

Extensive studies have documented the aberrant expression of GPX3 in patients with various cancers, highlighting its involvement in tumor cell proliferation, adhesion, migration and metastasis. The decreased expression of GPX3 in tumor tissue correlates with increased tumor cell proliferation and invasion, and poor prognosis, positioning GPX3 as a potential diagnostic marker and therapeutic target for tumors [23,24,25]. However, the significance of GPX3 in non-neoplastic diseases also merits attention, as emerging research underscores its pivotal role in these conditions. This review aims to summarize the latest insights into GPX3 expression and function, discuss its regulation and emphasize its potential as a novel therapeutic target in noncancerous diseases. Since the implications of GPX3 in cancer have been well documented and extensively reviewed [23,24], this issue is beyond the scope of this paper, and this paper will focus primarily on its roles outside of oncology.

## 4. Research Progress in GPX3 and Non-Neoplastic Diseases

Paragraph content.

**Figure 3:**
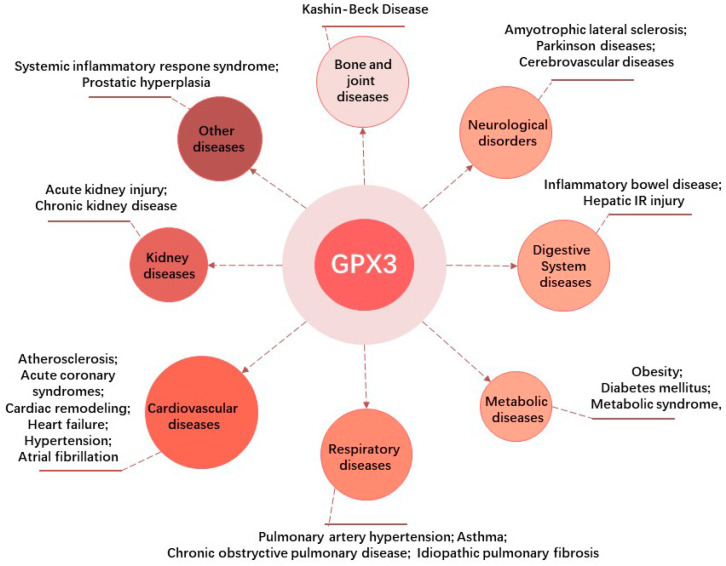
Involvement of GPX3 in non-neoplastic disorders. The figure was created with Figdraw (www.figdraw.com, accessed on 1 June 2024).

The expression and activities of GPX3 are changed in many pathological processes involved in systemic non-neoplastic disorders, such as kidney diseases, cardiovascular diseases, respiratory diseases, metabolic diseases, digestive system diseases, neurological disorders, bone and joint diseases, and other diseases (Figure 3). 

### 4.1. GPX3 and Kidney Diseases

The kidney is a vital organ responsible for maintaining homeostasis in various bodily functions [44]. Kidney disease, including acute kidney injury (AKI) and chronic kidney disease (CKD), presents significant global health challenges, and its prevalence has increased rapidly in recent years [45]. Oxidative stress has been reported in kidney disease due to both antioxidant depletion and increased ROS production, which can accelerate kidney disease progression [46]. Thus targeting oxidative stress may be a promising therapeutic approach.

GPX3 is abundantly expressed in the kidneys, synthesized specifically in renal tubular cells and released into circulation or bound to tubular basement membranes [16].

#### 4.1.1. AKI

AKI, marked by rapid deterioration of kidney function and reduced urine output, has high morbidity and mortality rates in clinical practice [47]. Renal ischemia-reperfusion (IR) injury, a common cause of AKI in clinical settings, is associated with increased oxidant stress.

GPX3 is notably downregulated in renal IR injuries and serves as a potent biomarker due to its pivotal involvement in oxidative stress, the immune response and apoptosis-related signaling pathways [48]. Studies have shown that the transcription of GPX3 is significantly reduced during the acute phase of renal IR injury in rats, perpetuating oxidative stress within renal tissues [49]. Wu et al. demonstrated a substantial decrease in GPX3 levels in renal tissues obtained from both IR-induced AKI mouse models and clinical AKI patients, with a positive correlation observed between GPX3 levels and the severity of renal injury. Their investigation revealed that upregulating GPX3 expression through the use of a GPX3 overexpression plasmid mitigated IR-induced oxidative stress. Additionally, agents such as vitamin D and its receptor agonists such as paricalcitol confer protection against IR-induced renal injury in part through the modulation of GPX3 expression. Consequently, the depletion of renal GPX3 may serve as a hallmark of renal oxidative stress injury, while the preservation of renal GPX3 represents a potential therapeutic avenue for IR-induced AKI [50]. Furthermore, Wu et al. noted a reduction in the concentration of the GPX3 isoform during renal IR injury, particularly within the cell membranes of proximal tubular epithelial cells. This observation suggests the critical role of GPX3, a vital selenoprotein, in safeguarding proximal tubular epithelial cell membranes against oxidative damage during renal IR injury [51]. Moreover, the reduction in GPX3 expression was mitigated by treatments such as apocynin and neutrophil deficiency, suggesting that enhancing GPX3 could be a strategic antioxidant defense against renal IR injury [52]. Furthermore, miR-483-5p was shown to exacerbate cisplatin-induced AKI by targeting GPX3, whereas viral-mediated overexpression of GPX3 prevented AKI by reducing oxidative stress and apoptosis in tubular cells [41]. Recombinant Klotho was also found to alleviate vancomycin-induced AKI through upregulating antioxidative capacity via the JAK2/STAT3/GPX3 pathway [53]. Serum GPX3 concentrations were significantly lower in patients in the cardiac surgery-associated AKI (CSA-AKI) group. The GPX3 ratio, the ratio of the preoperative and 6-h postoperative GPX3 protein concentrations, has good predictive value for CSA-AKI and may be a potential early diagnostic marker for AKI [54].

#### 4.1.2. CKD

CKD, characterized by a progressive decline in renal function, leads to end-stage renal disease and poses a severe public health issue globally [55]. Oxidative stress is implicated in both the progression of CKD and its associated complications [56].

GPX3 expression is downregulated during CKD, and this deficiency significantly contributes to the disease’s pathophysiology. In vivo studies have shown that GPX3 knockdown increases extracellular matrix expression and exacerbates kidney fibrotic lesions following obstructive injury. Conversely, exogenous overexpression of GPX3 reduces kidney fibrosis and inhibits NADPH oxidase 2 and p38 mitogen-activated protein kinase activity [27]. Reduced GPX3 expression in the kidneys of CKD mouse models triggers NOX4 mRNA and protein expression, leading to increased oxidative stress and fibroblast proliferation and activation [57]. CKD patients typically exhibit low GPX3 levels, closely associated with the development of kidney disease-induced cardiac complications [58]. Furthermore, an inverse relationship exists between GPX3 activity and the rate of eGFR decrease in patients with diabetes and advanced CKD. Antioxidant treatment in patients with type 2 diabetes and early CKD stages can derepress renal blood flow and improve the eGFR, directly correlated with GPX3 activity. Notably, after 12 months of follow-up, patients receiving usual care with higher GPX3 activity maintained improved renal function [59].

### 4.2. GPX3 and Cardiovascular Diseases (CVDs)

CVDs, including ischemic heart disease, stroke, heart failure, peripheral arterial disease, and other cardiac and vascular conditions, are the primary cause of mortality worldwide and significantly impair quality of life [60]. Oxidative stress plays a crucial role in cardiac pathophysiology, where ROSs activate myocardial hypertrophic signaling kinases and transcription factors, leading to matrix remodeling, cellular dysfunction, and ultimately cardiac dysfunction [61,62].

**Figure 4:**
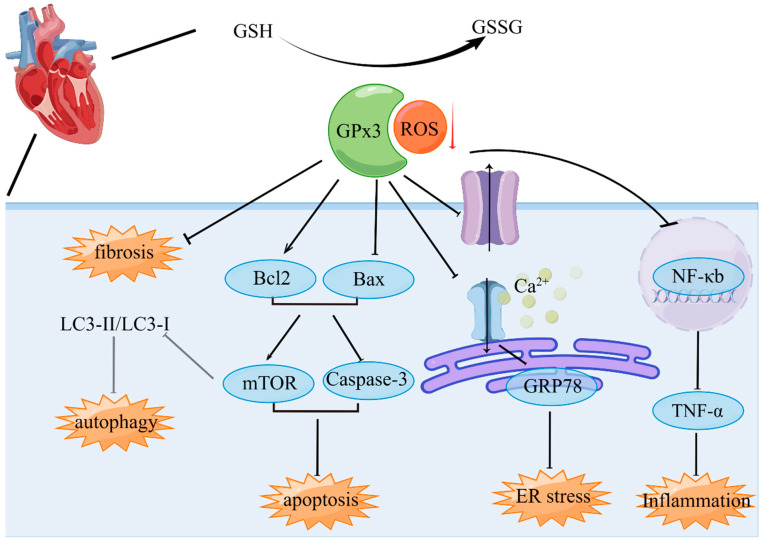
Effects of GPX3 on cardiovascular diseases. GPX3 plays a protective role in cardiovascular diseases by reducing fibrosis, autophagy and apoptosis through the mTOR and Caspase-3 pathways, preserving intracellular Ca^2+^ homeostasis and ER stress, and inhibiting inflammatory responses via the NF-κb/TNF-α pathway. ER: Endoplasmic reticulum; GSH: glutathione; GSSG: oxidized glutathione disulfide. The figure was built using Figdraw (www.figdraw.com, 1 June 2024).

Elevated levels of GPX3 have been associated with protective effects against cardiac injury [63]. A prospective study by Buijsse et al. reported lower GPX3 activity in individuals who died from CVDs compared to controls, revealing a linear and inverse relationship between serum GPX3 activity and CVDs mortality in individuals with low HDL, encompassing conditions such as coronary heart disease, other atherosclerotic diseases, and stroke [64]. The molecules involved in GPX3 inhibition-induced myocardial injury include those regulating autophagy, apoptosis, Ca^2+^ homeostasis, endoplasmic reticulum stress, and inflammatory responses [65,66] (Figure 4). However, the detailed mechanisms of GPX3 in cardiovascular diseases remain to be fully elucidated.

#### 4.2.1. Atherosclerosis

Atherosclerosis, a chronic inflammatory disease of the vascular system, is a major cause of severe vascular events such as coronary artery disease (CAD), myocardial infarction (MI), stroke, and peripheral artery disease. Excessive ROS production leads to oxidative stress, a significant risk factor for the initiation and progression of atherosclerosis [67,68].

Studies have noted decreased GPX activity in the serum of patients with CAD compared to control subjects, but the authors did not further investigate the activity of specific GPX subtypes [69]. Research by Jin et al. on GPX3 knockout mice revealed an 80% reduction in plasma GPX3 activity, which was associated with increased platelet activation, vascular dysfunction, and increased platelet-dependent arterial thrombosis [70]. This finding suggested that GPX3 is a crucial mediator in conditions that may impair endothelial function and lead to atherosclerosis, as evidenced by vascular endothelial damage in GPX3-deficient mice [71]. However, how GPX3 induces vascular endothelial damage remains to be investigated.

#### 4.2.2. Acute Coronary Syndrome (ACS)

Despite declining incidence rates, ACS, which includes unstable angina and acute MI, is a leading cause of premature death [72]. Oxidative stress plays a pivotal role in the pathogenesis of ACS [73,74]. Studies have shown that GPX3 activity, along with its protein and mRNA levels in the plasma, is significantly elevated in ACS patients compared to those with stable CAD and healthy controls, and these increased levels of GPX3 are associated with improved outcomes [75].

MI is the most common form of ACS, and blockage of a coronary artery due to blood clotting leads to ischemia and subsequent cell death [76]. Kumar et al. reported that GPX3 is upregulated in AMI, suggesting its potential for developing therapeutic strategies for managing acute MI [63]. Furthermore, revascularization by coronary artery bypass graft surgery (CABG) is effective in relieving MI symptoms and decreasing mortality [76]. Postoperative atrial fibrillation (POAF), a common complication following CABG surgery, has been linked to increased GPX3 activity, indicating that GPX3 may be developed as a biomarker for predicting POAF [77,78]. In addition, the regenerative potential inherent in the neonatal heart presents promising avenues for MI therapy. Liu et al. elucidated the upregulation of GPX3 in MI and delineated its candidacy as a prospective target for heart regeneration therapy through an integrative approach encompassing transcriptomic and proteomic analyses [79].

#### 4.2.3. Pressure Overload Induced Cardiac Remodeling

Cardiac remodeling, a process associated with conditions such as hypertension, MI and valvular disease, involves changes in the heart’s structure and function in response to chronic stress and injury [80,81,82]. Oxidative stress is a key regulator of pressure overload induced cardiac remodeling [83]. Investigations suggest that GPX3 may participate in the modulation of myocardial remodeling under pressure loading.

Li et al. observed t a significant impairment in oxidative stress resistance in mice following transverse aortic coarctation. They noted a negative correlation between GPX3 expression levels and fibrosis levels across various treatment groups of the bromodomain inhibitor JQ1, suggesting a potential role of GPX3 in promoting the differentiation of cardiac fibroblasts into a protective state. This mechanism may mitigate myocardial fibrosis and inhibit oxidative stress under pressure overload [28]. Moreover, Covington et al. uncovered that GPX3 deficiency exacerbates maladaptive right ventricular remodeling and leads to right ventricular dysfunction in experimental models of pulmonary artery banding (PAB). This exacerbation was evidenced by increased right ventricle expression levels of connective tissue growth factor, transforming growth factor-β and atrial natriuretic peptide in GPX3-deficient PAB animals [84].

#### 4.2.4. Heart Failure (HF)

HF, characterized by structural and functional cardiac changes, includes ischemic cardiomyopathy (ICM) and dilated cardiomyopathy (DCM). The role of oxidative stress in HF is well supported by the literature, suggesting that antioxidant enzymes could mitigate heart failure triggers [85,86]. The overexpression of antioxidant enzymes protects the heart from a variety of heart failure inducers [87].

Patients with ICM exhibit significantly lower mean plasma and platelet GPX activities [88]. Similarly, Lu et al. noted decreased GPX3 expression in HF induced by DCM and ICM [89]. Choi et al. proposed a statistical learning framework for predicting left ventricular ejection fraction based on GPX3 levels in the ICM and reported that higher GPX3 levels (≥5.314 μg/mL) were associated with reduced left ventricular ejection fraction (<50%) [90].

#### 4.2.5. Hypertension

Hypertension is a well-established independent risk factor that predisposes individuals to fatal complications in CVDs. Oxidative stress is recognized as a key driver of endothelial damage and vascular stiffness, which are fundamental contributors to hypertension and other cardiovascular conditions [91].

Research has indicated an association between GPX3 gene polymorphisms, specifically rs3828599, and hypertension in a rural Han Chinese cohort [92]. However, this association was not replicated in a larger Japanese cohort [93]. In Thai populations, the GPX3 rs3828599-GG variant was linked to the incidence of hypertension [94]. Additionally, transcriptomic analysis of small arteries from hypertensive patients with chronic kidney disease revealed that miR-338-3p targets GPX3. Downregulation of miR-338-3p could lead to upregulation of GPX3, suggesting its potential as a novel therapeutic target for managing hypertension and vascular damage in CKD patients [95].

#### 4.2.6. Atrial Fibrillation (AF)

AF is the most common sustained cardiac arrhythmia in clinical practice, and its incidence significantly increases with age [96]. AF is characterized by an oxidative imbalance, and accumulating evidence has implicated oxidative stress in its pathogenesis [97].

Pastori et al. demonstrated that a decrease in GPX3 with age increases the risk of cardiovascular events in individuals with AF, indicating that decreased GPX3 activity may serve as a predictor of both fatal and nonfatal cardiovascular complications [98]. The Mediterranean diet has been shown to favorably modulate the antioxidant activity of GPX3 in AF, thereby reducing the rate of vascular events. This suggests that dietary modulation of GPX3 levels could be a viable strategy to prevent cardiovascular incidents in AF patients [99]. Additionally, Menichelli et al. reported that elevated levels of circulating lipopolysaccharides, which may impair antioxidant status, are correlated with reduced GPX3 activity and increased risk of cardiovascular events in AF patients [100]. In addition, as mentioned above, patients with POAF following CABG have increased GPX3 activity, and GPX3 may be developed as a biomarker to predict POAF [77,78].

### 4.3. GPX3 and Respiratory Diseases

The lungs, constantly exposed to a highly oxidizing environment, have evolved various mechanisms to mitigate oxidative stress [101], which prominently features in respiratory diseases and exacerbates conditions such as asthma, chronic obstructive pulmonary disease (COPD) and infections [102].

In lung tissue, GPX3 is expressed within bronchial epithelial cells and mesenchymal fibroblasts, predominantly localized along the basement membrane of the bronchial epithelium, endothelium, endothelium, and extracellular matrix [103]. As a pivotal antioxidant in the pulmonary milieu, GPX3 expression is modulated across a spectrum of respiratory pathologies [104].

#### 4.3.1. Pulmonary Artery Hypertension (PAH)

PAH is characterized by elevated pulmonary artery pressure and increased pulmonary vascular resistance, which can lead to maladaptive right ventricular remodeling, heart failure, and even death [105,106]. Anti-oxidative treatment that attenuates PAH-induced ROS production has been shown to improve right ventricular function [107].

Patients with PAH have lower transcriptional levels of GPX3 in lung tissues, identifying GPX3 as a hypoxia-induced metabolism-associated hub gene, which could provide insight into the molecular mechanisms of hypoxic PAH and potential therapeutic targets [108]. Systemic sclerosis-related PAH is the final presentation of progressive pulmonary vasculopathy. Researchers have found that systemic sclerosis-related PAH patients also exhibit lower serum levels and activity of GPX3 than healthy controls [109].

#### 4.3.2. Asthma

Asthma, a common chronic inflammatory airway disease, is associated with increased ROS production [110]. Oxidative stress is particularly elevated in severe asthma and during exacerbations, and is exacerbated by air pollution, which promotes airway inflammation and hyper-responsiveness [111].

Research has shown that plasma GPX levels are significantly lower in asthmatic patients than in healthy individuals [112]. Moreover, a different study confirmed a significant decrease in GPX activity in patients with asthma [113]. Microarray expression analyses and bronchial biopsy evaluations conducted in asthma patients and healthy controls have revealed the downregulation of GPX3 expression in asthma patients [114]. Additionally, genetic studies have identified GPX3 rs2070593 as a protective locus against asthma development, with two allelic mutations in GPX3 rs2070593 preventing the development of asthma [115].

#### 4.3.3. COPD

COPD, characterized by chronic inflammation, alveolar destruction (emphysema) and bronchiolar obstruction, is significantly influenced by oxidative stress [116].

Studies have demonstrated decreased GPX activity and expression in the erythrocytes/plasma of COPD patients [117,118]. A study by Reddy et al. showed that both the expression and activity of GPX3 were reduced in the lung tissues of COPD patients and in normal human bronchial epithelial cells treated with cigarette smoke extract (CSE) [119]. The underlying mechanism might be that CSE reduces GPX3 expression by downregulating the expression or activity of PPARγ [119]. However, a meta-analysis did not find significant differences in GPX3 levels between COPD patients and controls, although the quality of the evidence was low [120]. Experiments exposing rats to NO2, which mimics the inflammatory response in human COPD, revealed increased GPX3 mRNA expression and activity in bronchoalveolar lavage fluid (BALF) [121]. These controversial findings might be related to differences in tissues or species, and further studies need to determine the underlying mechanisms involved.

#### 4.3.4. Idiopathic Pulmonary Fibrosis (IPF)

IPF is a fatal and irreversible interstitial lung disease characterized by the involvement of ROSs in specific fibrotic processes, including macrophage polarization, immunosenescence, alveolar epithelial cell apoptosis, myofibroblast differentiation, and alterations in the acellular extracellular matrix [101]. Recent studies have highlighted the therapeutic potential of ROS-responsive liposomes in IPF, suggesting that targeting oxidative stress could form a basis for novel therapeutic intervention [101,122,123].

In vivo models of IPF induced by bleomycin, a chemotherapeutic agent known to cause pulmonary fibrosis via ROS production, have shown a significant reduction in GPX levels in treated groups compared to controls [124,125]. Research by Zeng et al. indicated that GPX3 expression is downregulated in the lung tissues of IPF patients, leading to increased oxidative stress and exacerbating the fibrotic phenotype [126]. Similarly, Chien et al. observed a decrease in GPX3 protein expression in lung tissues following bleomycin treatment [127]. In contrast, Schamberger et al. reported that GPX3 was upregulated in lung homogenates from IPF patients and in mouse bronchoalveolar lavage fluid (BALF) during bleomycin-induced lung fibrosis, suggesting that GPX3 is expressed by bronchial epithelial cells and secreted in its active form into the epithelial lining fluid [103]. These conflicting findings could be attributed to variations in the source of GPX3, whether derived primarily from the kidneys or locally expressed and secreted by bronchial epithelial cells. Further studies with larger patient cohorts are necessary to clarify the underlying mechanisms involved.

#### 4.3.5. Other Respiratory Diseases

Exposure to hyperoxia for 72 h has been shown to increase GPX3 protein levels in both the plasma and lungs of mice, suggesting a protective response to oxidative stress [128]. He et al. identified GPX3 as a potential diagnostic biomarker for patients with hypersensitivity pneumonitis [129]. Additionally, Marko Markovic revealed a significant association between the *GPX3* rs8177412 variant genotype and the risk of developing severe forms of COVID-19, suggesting that GPX3 is a complementary diagnostic tool for predicting the disease course [130].

### 4.4. GPX3 and Metabolic Disorders

Metabolic disorders, including obesity, diabetes, and insulin resistance, pose significant public health challenges due to their high associated morbidity and mortality rates [131]. There is a growing body of evidence linking increased oxidative stress to the pathogenesis of these disorders [132,133].

#### 4.4.1. Obesity

Obesity, characterized by the abnormal or excessive expansion of white adipose tissue, is now recognized as a global pandemic and a critical health concern [134]. Oxidative stress plays a central role in the pathophysiology of obesity, contributing to adipose tissue dysfunction and serving as a significant source of ROSs [132,135].

Initial studies identified GPX3 expression in human adipose tissue as early as 1997 [136]. However, findings regarding the relationship between GPX3 expression and obesity remain mixed. For instance, GPX3 expression was found to be elevated in the serum of overweight and obese individuals in central Mexico, with serum GPX3 concentration positively correlating with body weight and inversely correlating with insulin sensitivity [137]. GPX3 rs922429 was shown to protect against obesity according to body fat percentage in a study in Mexico [138]. Conversely, other studies reported no significant differences in GPX3 serum concentrations between obese and lean individuals, although GPX3 expression in subcutaneous adipose tissue increased after weight loss and was greater in lean and insulin-sensitive individuals than in their obese and insulin-resistant counterparts [139]. Lee et al. showed that GPX3 was highly expressed in adipose tissue, and its expression was reduced in both the serum and adipose tissue of obese subjects, attributing this reduction to pro-oxidant conditions such as hypoxia and inflammation [140]. Some researchers believe that adipose tissue GPX3 expression significantly correlates with age, BMI, fat distribution, and insulin sensitivity but not with circulating GPX3 [139]. However, others believe that circulating GPX3 levels are closely correlated with adipose GPX3 expression [140]. These discrepancies in findings suggest that the relationship between circulating GPX3 levels and adipose GPX3 expression warrants further investigation.

#### 4.4.2. Diabetes Mellitus (DM)

DM is a metabolic disease characterized by long-term hyperglycemia, leading to serious complications such as microangiopathy and macroangiopathy, significantly increasing mortality [141]. Oxidative stress is a major pathogenic factor in diabetes and contributes to endothelial dysfunction by reducing nitric oxide availability. The role of oxidative stress in the occurrence and development of diabetes mellitus is both critical and pivotal [142].

Integrative analysis of multiple diabetes genome anatomy project datasets identified GPX3 as one of the most differentially expressed genes [143]. However, studies have shown that GPX3 mRNA expression is significantly lower in the adipose tissue of type 2 diabetes (T2D) patients than in individuals with normal glucose metabolism [139]. In contrast, Iwata et al. reported that GPX3 expression is upregulated in the hearts of mice under hyperglycemic conditions and plays a crucial role in protecting cardiomyocytes from hyperglycemia-induced oxidative stress in the cardiac tissue of diabetic mice [144]. Multiple investigations have demonstrated a negative correlation between the activity of GPX3 and the development of carotid atherosclerotic plaques, suggesting that diminished GPX3 activity could serve as an autonomous prognostic indicator for carotid atherosclerosis in individuals with type 2 diabetes mellitus (T2DM) [145,146]. Furthermore, Demircan et al. reported that lower GPX3 activity during both the early and late stages of pregnancy is independently linked to a greater risk of gestational DM and greater gestational age [147]. The observed heterogeneity in GPX3 expression across these investigations is markedly pronounced. Discrepancies in ethnicity, concurrent pharmacotherapy and dietary patterns within human cohorts may underpin the diverse associations between GPX3, obesity, and T2D.

Common therapeutic approaches for DM, including oral hypoglycemics and exogenous insulin replenishment, can influence GPX3 expression. For instance, in an obese mouse model, the use of antioxidants and rosiglitazone was found to increase GPX3 expression [39,140]. Conversely, troglitazone was shown to inhibit GPX3 expression in rat adipose tissue [148]. Thiazolidinediones (TZDs), by activating PPAR, induce GPX3 expression, which in turn reduces extracellular H_2_O_2_ levels and modulates insulin resistance. Silencing GPX3 essentially nullifies the antioxidant effects of TZDs, indicating that the PPAR-dependent antioxidant effects of these drugs are mediated through GPX3 [39].

#### 4.4.3. Metabolic Syndrome

Metabolic syndrome encompasses a cluster of typical cardiovascular risk factors, including obesity, insulin resistance, hypertension, and dyslipidemia [149]. This syndrome is also known as “insulin resistance syndrome”, as a substantial part of its pathophysiology is driven by resistance to the metabolic effects of insulin [150]. Patients diagnosed with metabolic syndrome exhibit increased delivery of ROSs, increased lipid peroxidation, and reduced antioxidant defense mechanisms. An increasing number of studies corroborate the pivotal role of oxidative stress in both the etiology and pathogenesis of metabolic syndrome [151,152].

**Figure 5:**
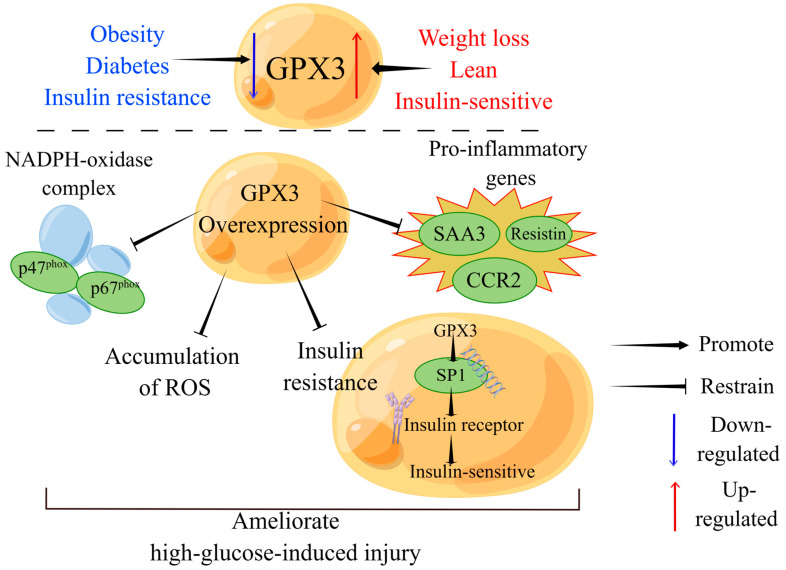
GPX3 expression in adipocytes and its potential pathways. Adipose GPX3 expression is down-regulated in obese, diabetic and insulin-resistant individuals, but up-regulated in weight loss, lean and insulin-sensitive individuals. Overexpression of GPX3 in adipocytes significantly reduced the expression of pro-inflammatory genes such as *SAA3*, *resistin* and *CCR2*, suppressed the expression of the p47 and p67 subunits of the NADPH-oxidase complex, reduced ROS buildup and attenuated insulin resistance. As a result, the harm caused by high-glucose is reduced. Furthermore, by activating the transcription factor SP1, GPX3 increases insulin receptor expression and adipose tissue insulin sensitivity. The figure was created with Figdraw (www.figdraw.com, 1 June 2024).

Recently, GPX3 has been recognized as a novel regulator of insulin receptor expression, insulin receptor and GPX3 expression were positively correlated in mouse models of obesity and insulin resistance: By activating the transcription factor SP1, GPX3 promotes insulin receptor expression in 3T3-L1 preadipocytes and improves adipose tissue insulin sensitivity. On the contrary, with dysregulation of GPX3 potentially impacting insulin receptor functionality and contributing to insulin resistance [26,153]. In the adipose tissue of insulin-resistant and obese patients, treatment with selenite was shown to enhance insulin receptor expression in 3T3-L1 pre-adipocytes, improving adipocyte differentiation and function through the induction of GPX3 expression and activation of the transcription factor SP1 [26]. In a study involving a Mexican cohort of metabolic syndrome patients, elevated serum GPX3 levels were correlated with decreased insulin sensitivity and cardiovascular risk (triglycerides/high-density lipoprotein-cholesterol index) [154]. Furthermore, overexpression of GPX3 in adipocytes significantly reduced the expression of pro-inflammatory genes such as *SAA3*, *resistin* and *CCR2* induced by high glucose levels and attenuated hyperglycemia-induced insulin resistance [140]. Additionally, it suppressed the expression of the p47 and p67 subunits of the NADPH-oxidase complex, reduced the accumulation of ROSs, and improved hyperglycemia-induced insulin resistance [140] (Figure 5).

### 4.5. GPX3 in Digestive System Diseases

Digestive system diseases, including inflammatory bowel disease (IBD), diverticulitis, colorectal cancer, and liver diseases, arise primarily through the interplay of genetic and environmental influences. There conditions pose a formidable challenge to human health and have become a growing global burden [155,156]. Oxidative stress is a major contributor to gastrointestinal mucosal diseases, with the overproduction of ROSs implicated in the development of these conditions [157,158].

GPX3 mRNA is primarily localized to mature absorptive epithelial cells in both the human and mouse large intestines. GPX3 can be synthesized and secreted into the external environment by binding to the basement membrane of epithelial cells in the gut and gastrointestinal tract [32,159]. GPX3 has been detected in the small intestine, cecum, and large intestine of rats, with the highest protein levels found in the cecum [159].

#### 4.5.1. IBD

IBD, including ulcerative colitis and Crohn’s disease, is characterized by chronic inflammation and remodeling of gastrointestinal tract tissues caused by a dysregulated immune response to the intestinal microbiota in genetically susceptible individuals [160]. Oxidative stress plays a crucial role in the pathogenesis of IBD, and increased levels of ROSs coupled with decreased levels of antioxidants contribute to disease pathogenesis [161,162].

Clinical studies have reported elevated GPX3 activity in the plasma of children with IBD [163,164]. Specifically, children with Crohn’s disease exhibit greater GPX and vitamin E concentrations than healthy controls, while plasma antioxidant concentrations are not significantly different between children with ulcerative colitis and healthy subjects [164]. In a dextran sodium sulfate-fed IBD mouse model, similar increases in plasma GPX activity were noted, comparable to those observed in human IBD patients [165]. Tham et al. suggested that inflammatory injury in the intestine might trigger the observed increase in plasma GPX activity, which is associated with increased GPX3 mRNA levels in the kidney [165]. Guo et al. synthesized polyethylene glycol-modified Mo_3_Se_4_ nano flakes (PMNFs) with multiple antioxidant enzymatic activities, including GPX3. These PMNFs activated the Nrf2-Keap1 antioxidant pathway, significantly reducing disease activity index scores and reversing sodium dextran sulfate-induced IBD [166]. Therefore, various antioxidant treatments, represented by GPX3, hold promising potential for IBD treatment strategies [166]. Additionally, GPX3 expression serves as a valuable biomarker for the early detection and progression to colorectal cancer [167].

#### 4.5.2. Hepatic IR Injury

Hepatic IR injury, a critical factor in liver damage during surgical procedures such as hepatic resection and liver transplantation, is a leading cause of graft dysfunction and liver failure post-transplantation [168]. During IR, the redox balance is disrupted, leading to the accumulation of ROSs, which are pivotal in the pathogenesis of hepatic IR injury [169]. Consequently, antioxidants are considered potential therapeutic agents.

Human induced pluripotent stem cell-derived mesenchymal stem cells (hiPSC-MSCs), known for their high proliferation rate and engraftment capacity, have been engineered to deliver GPX3 [170]. This intervention has been shown to ameliorate hepatic IR injury by inhibiting hepatic senescence, reducing hepatic apoptosis, and promoting liver regeneration [171]. Recombinant GPX3 (rGPX3) has been shown to inhibit the cellular senescence of liver cells in a dose-dependent manner, with genes such as *CD44*, *Nox4*, *IFNG*, and *SERPERINB2* identified as mediators of the suppressive effects of GPX3 on hepatic senescence [171]. Treatments that enhance hepatic GPX3 activity, such as paeoniflorin, have been shown to attenuate hepatic IR injury [172,173]. Therefore, modulating the dysregulated micro-environment and enhancing GPX3 supplementation could be viable strategies for addressing liver IR injury.

Moreover, the prevalence of alcoholic liver disease, a common outcome of prolonged and heavy alcohol intake and a leading cause of chronic liver disease worldwide, is also influenced by GPX3 levels. Li et al. discovered that upregulating GPX3 offers protection against alcohol-induced hepatic injury in mice [174]. Notably, a micro-environment with reduced GPX3 expression can accelerate cellular senescence, leading to significant inflammation and severe liver graft injury [175]. Furthermore, in paclitaxel-induced hepatorenal toxicity in rats, GPX3 expression was significantly downregulated [176].

### 4.6. GPX3 and Neurological Disorders

Neurological disorders, including Alzheimer’s disease, Parkinson’s disease (PD), Huntington’s disease, amyotrophic lateral sclerosis (ALS), stroke, hypoxic-ischemic brain injury, epilepsy, and traumatic brain injury, place a substantial burden on families and society [177]. The mechanisms underlying neurological disorders are complex, and oxidative stress is recognized as a critical regulatory factor, making it a focus of current research [178,179]. Increasing evidence highlights the significant role of GPX3 in the pathogenesis of developmental, degenerative, and behavioral neurological disorders. GPX3 has been identified as a genetic risk locus shared among Alzheimer’s disease and related dementias, PD, and ALS [180].

#### 4.6.1. Amyotrophic Lateral Sclerosis (ALS)

ALS is a degenerative disorder characterized by muscle weakness due to motor neuron degeneration, and its underlying mechanism is not yet clear [181,182]. The pathogenesis of ALS is influenced by various external environmental factors, such as exposure to chemicals, metals, and pesticides, which interact with internal susceptibility factors to contribute to the disease. Recent studies have linked oxidative stress to the pathogenesis of ALS [183].

Genome-wide association studies have identified 10 risk loci for ALS, including GPX3 on chromosome five, suggesting that GPX3 is a lead ALS risk gene [184]. Wray and colleagues highlighted the association of the GPX3-*TNIP1* locus with ALS using cross-ethnic meta-analyses [185]. A comparative study noted no difference in the plasma GPX3 concentration between ALS patients and controls; however, GPX3 activity was significantly lower in ALS patients [186]. Tanaka et al. reported that GPX3 protein levels in ALS model rats carrying the mutant superoxide dismutase-1 (SOD1)^H46R^ initially increased during the pre-symptomatic stage but decreased as the disease progressed [187]. This pattern suggests that GPX3 could serve as a serum biomarker for ALS and is useful for detecting disease presence and monitoring its progression [187]. Furthermore, Mendelian randomization analyses have provided genetic evidence supporting the therapeutic targeting of GPX3 in ALS treatment [188].

#### 4.6.2. Parkinson’s Disease (PD)

PD, a progressive motor neurodegenerative disorder, is the second most common neurodegenerative disease among elderly people [189]. Oxidative stress plays a critical role in the pathogenesis of PD by influencing various enzymes and signaling molecules [190].

Duke et al. reported increased expression of GPX3 in the medial nigral component of individuals with PD, suggesting a compensatory response to increased oxidative stress [191]. Additionally, Jiang et al. reported that GPX3 expression was upregulated in PD patients, suggesting that GPX3 is a potential biomarker for this disease [192].

#### 4.6.3. Cerebrovascular Diseases

Cerebrovascular diseases, including ischemic and hemorrhagic strokes, aneurysms and vascular malformations, are caused by problems with the brain vasculature and are associated with high morbidity and mortality [193]. ROSs are crucial for the onset and progression of cerebrovascular diseases [194].

GPX3 is essential for maintaining vascular redox homeostasis, and its deficiency has been linked to thrombotic disorders and familial stroke [195,196]. Decreased GPX3 activity in the plasma of patients with cerebral thrombotic disorder leads to high ROS levels and rapid nitric oxide inactivation, whereas exogenous GPX3 supplementation in patients’ plasma can reverse nitric oxide-mediated platelet inhibition, offering protection against thrombotic disorder [195]. Furthermore, in vitro experiments utilizing eight single-nucleotide polymorphisms in plasma revealed a GPX3 promoter H2 polymorphism, which was identified as a risk factor for arterial ischemic stroke and cerebral venous thrombosis in a limited cohort of young Brazilian adults. Notably, this polymorphism reduced hypoxia-induced GPX3 transcription [197,198]. Single-nucleotide polymorphisms (SNPs) in the promoter region of the GPX3 gene have been associated with ischemic stroke risk in various populations, including Caucasians and young Asian Indians [199]. However, Nowak-Göttl et al. reported that GPX3 genetic variants are risk factors for arteriopathy-related stroke in children but not for thromboembolic stroke or cerebral sinovenous thrombosis [200]. These findings underline the potential of targeting GPX3 for preventing and treating arterial thrombotic disorders [201].

### 4.7. GPX3 and Bone and Joint Diseases

Bone and joint diseases, such as osteoporosis, fracture healing, osteoarthritis, inflammatory arthritis, and bone metastasis, are influenced by oxidative stress, a key modulator of cell fate decisions in osteoarthritis and osteoporosis [202,203].

#### Kashin-Beck Disease (KBD)

KBD is an osteoarticular disease that affects the epiphyseal plates of children before the epiphysis closes, leading to permanent disability [204]. Selenium deficiency is a well-recognized risk factor for KDB [205].

Studies have shown significantly reduced GPX3 mRNA levels in chondrocytes from KBD patients, where elevated methylation of CpGs in the GPX3 gene suppresses its transcription, reducing antioxidant function and promoting chondrocyte apoptosis, thus accelerating KBD progression [205,206]. Selenium supplementation can reverse this methylation status, increasing GPX3 expression and inhibiting the PI3K/Akt/c-fos signaling pathway, thereby exerting a protective effect [206].

Additionally, increased GPX3 expression has been observed in bone samples from hip fracture patients compared to controls, suggesting increased anti-oxidative activity in bone samples from elderly osteoporotic women with hip fractures [207]. Auranofin, an anti-arthritic drug that has been used clinically for many years, has been shown to significantly influence GPX3 activity, with potential medical implications [208].

### 4.8. GPX3 and Other Diseases

GPX3 plays a significant role in inflammatory responses. Decreased GPX3 activity has been observed in individuals with systemic inflammatory response syndrome, providing insight into this condition. Plasma GPX3 levels are markedly lower in patients with multiple organ failure and systemic inflammatory responses [209]. Additionally, functional impairment of GPX3 contributes to increased ROS production and is implicated in the pathogenesis of prostatic hyperplasia [210].

## 5. Conclusions

As research on ROSs has advanced, an increasing number of diseases have been found to be linked to oxidative stress. Early studies on antioxidant therapy largely ended in failure and did not advance to clinical application. GPX3, an essential member of the GPX family and a critical component of the cellular antioxidant system, remains a key first line of defense against harmful ROSs. Numerous basic and clinical studies have confirmed the role of GPX3 in both physiological functions and pathological processes. This article reviews current advances in GPX3 research within non-neoplastic diseases, offering insights into its functions and mechanisms. Given its established roles across various fields and its strong association with the pathogenesis of diverse diseases, including kidney diseases, respiratory diseases and cardiovascular diseases, understanding the molecular role of GPX3 may provide valuable insights for predicting disease prognosis and identifying new therapeutic targets for diagnostic and treatment strategies. However, the precise mechanisms of GPX3 action remain unclear, and related research is still in its infancy. Further studies are necessary to comprehensively elucidate the molecular role of GPX3, which could lead to novel diagnostic and treatment strategies.

(1) Variability in GPX3 expression: GPX3 expression varies across different tissues and in response to diverse oxidative stress conditions (Table 2). These disparities likely stem from differences in experimental animal models, tissue-specific locations, distinct cellular phenotypes responsive to oxidative stimuli, diverse pathophysiological mechanisms underlying diseases or tissue-specific regulatory pathways. For example, heightened GPX3 expression may result from the activation of antioxidant systems in response to oxidative damage [165], whereas diminished GPX3 expression often correlates with gene methylation and microRNA-mediated regulation [40,42,43].
(2) Diagnostic potential of GPX3: As an extracellular protein, GPX3 is readily detectable in blood, making it a promising biomarker for diagnosing diseases, assessing disease progression, and informing therapeutic decisions, for conditions such as cancer, IBD, inflammatory diseases, and ALS. Nevertheless, the limitation of using GPX3 as a biomarker is its credibility. Current studies are limited to animal models and small numbers of clinical samples, and GPX3 expression can be influenced by medications, diet and lifestyle. Expanding GPX3 testing in larger cohorts and diverse biospecimen repositories could validate its reliability as a biomarker.
(3) Therapeutic targeting of GPX3: Targeting GPX3 has shown promise in preclinical models (Table 3). Developing treatments to enhance GPX3 expression could extend to other diseases characterized by low GPX3 levels. However, there are currently no suitable medicines or agonists that can directly induce GPX3 expression, and many researchers have constructed overexpression plasmids to increase GPX3 expression in cells [50]. Further research is needed to develop new drugs based on stimulators or inhibitors that affect GPX3 expression, the enzyme itself and the involved receptors, and signaling pathways.

Despite the growing body of research related to GPX3, many unknowns remain. Ongoing investigations into the functions and regulatory mechanisms of GPX3, its role in biology, and its role in the diagnosis and treatment of diseases will deepen our understanding of its biological and medical significance. While transitioning from basic science to clinical applications presents challenges, focused mechanism-based research may unlock new diagnostic and therapeutic opportunities.

## Figures and Tables

**Table 1:** Characteristics of human glutathione peroxidases.

GPX Type	Peroxidatic Residue	Gene Location	Tissue Distribution	Cellular Localization	Reference
GPX1	Sec	3p21.31	Ubiquitously expressed; high in kidneys, liver, erythrocytes	Cytosol, peroxisome, mitochondrion	[13]
GPX2	Sec	14q23.3	Intestinal and pulmonary epithelium,	Cytosol	[14]
GPX3	Sec	5q33.1	Plasma, kidneys, adipose tissue, extracellular body fluids	Extracellular space	[15,16]
GPX4	Sec	19p13.3	Fat, testis, spermatozoa	Cytosol, nucleus, mitochondrion	[17]
GPX5	Cys	6p22.1	Testis, prostate, epididymis, spermatozoa	Extracellular	[18]
GPX6	Sec	6p22.1	Embryos, olfactory epithelium	Extracellular	[19]
GPX7	Cys	1p32.3	Placenta, thyroid, urinary bladder	Extracellular, endoplasmic reticulum	[20]
GPX8	Cys	5q11.2	Placenta, endometrium, ovary	Endoplasmic reticulum	[21]

Notes: Sec: Selenocysteine; Cys: Cysteine.

**Table 2:** Summary table of the expression and roles of GPX3, and the GPX3-involved signaling pathways in diseases.

Disease	Expression of GPX3 in Disease	Pathway Regulated	Roles of GPX3	Reference
AKI (IR induced)	Down in renal tissue	Oxidative stress; Apoptosis and immune responses	As a biomarker related to oxidative stress during renal IR injuries; Related to immune infiltration	[48]
AKI (IR induced)	Down in renal tissue	Oxidative stress	Positive correlation between GPX3 levels and the severity of renal injury	[49]
AKI (IR induced)	Down in renal tissue	Oxidative stress; Apoptosis	Loss of renal GPX3 may promote renal oxidative stress injury	[50]
AKI (IR induced)	Down in renal tissue	Oxidative stress	Protects proximal tubular epithelial cell membrane from oxidative damage in renal IR injury	[51]
AKI (IR or vancomycin induced)	Down in renal tissue	-	-	[52,53]
AKI (cardiac surgery associated)	Down in the serum	-	GPX3 ratio has predictive value for cardiac surgery associated AKI	[54]
CKD	Down in renal tubules	NOX4 upregulation in the extracellular microenvironment leads to fibroblast activation	Loss of *GPX3* mediates fibroblast activation via an oxidatively stressed extracellular microenvironment	[27]
CKD	Down in the kidney	Triggers NOX4 expression, oxidative stress and fibroblasts proliferation and activation	Orchestrates an oxidatively stressed extracellular microenvironment	[57]
CKD	Down in the kidney	-	GPX3 deficiency contributes to kidney disease-induced cardiac disease	[58]
Atherosclerosis	-	-	GPX3 deficiency promotes platelet-dependent thrombosis and enhances arterial thrombotic risk	[70]
ACS patients	Elevated in plasma (activity, protein, mRNA)	-	Higher levels of GPX3 associated with improved outcomes	[75]
AMI patients	Upregulated in blood	-	GPX3 may help develop therapeutic strategies for acute MI management	[63]
POAF following CABG	Increased in plasma	-	GPX3 may serve as biomarkers to predict POAF	[78]
MI (Mouse model)	Upregulated in myocardial tissue	-	GPX3 as a potential target for heart regeneration therapy	[79]
Cardiac remodeling	-	Inhibited oxidative stress	GPX3 promotes differentiation of cardiac fibroblasts into a protective state, attenuating myocardial fibrosis	[28]
HF (DCM and ICM)	Decreased in hearts	-	Higher serum GPX3 levels (≥5.314 μg/mL) closely related to reduced LVEF (<50%)	[89,90]
Hypertensive patients with CKD	Upregulated in gluteal subcutaneous small arteries	-	GPX3 could represent novel therapeutic targets for hypertension and vascular damage in CKD	[95]
Aging	Decline in serum	-	GPX’s decline increases the risk of cardiovascular events in individuals with atrial fibrillation	[98]
PAH	Lower in lung tissues	GPX3 is a hypoxia-induced metabolism-associated hub gene	Provides insight into the molecular mechanisms of hypoxic PAH and potential therapeutic targets	[108]
Systemic sclerosis related PAH	Activity is reduced in serum	-	-	[109]
Asthma	Downregulated in bronchial biopsies tissue	-	-	[114]
COPD (Patients and CSE-treated cells)	Reduced in human bronchial epithelial cells.	CSE reduces GPX3 expression by downregulating PPARγ expression or activity	GPX3 as a PPARγ transcriptional target, provides valuable information for developing more effective therapeutics for COPD.	[119]
COPD patients	No significantly difference in serum/plasma GPX3	-	-	[120]
COPD (Rats exposed to NO2)	Increased mRNA and activity in bronchoalveolar lavage fluid	-	-	[121]
IPF patients	Reduced in lung tissues	Oxidative stress	Influence the fibrotic phenotype	[126,127]
IPF patients	Upregulated in lung homogenates	-	-	[103]
IPF (Bleomycin- induced lung fibrosis)	Upregulated in mouse bronchoalveolar lavage fluid	-	-	[103]
Obesity	Elevated in the serum in central Mexico		Serum GPX3 concentration positively correlates with body weight and inversely with insulin sensitivity	[137]
Obesity	No difference in serum concentrations, lower in subcutaneous adipose tissue.	-	GPX3 higher in lean and insulin-sensitive individuals compared to obese and insulin-resistant counterparts	[139]
Obesity	Downregulated in plasma and adipose tissue	-	-	[140]
Diabetes mellitus (type 2 patients)	Lower mRNA in adipose tissue	-	-	[139]
Diabetes mellitus (mice)	Upregulated in hearts	Oxidative stress	Plays an important role in protecting cardiomyocytes	[144]
Metabolic syndrome patients	elevated in serum	-	Elevated GPX3 levels correlated with low insulin sensitivity and cardiovascular risk	[154]
IBD (children)	Increased activity in plasma	-	-	[163,164]
IBD mouse (dextran sodium sulfate-fed)	Increased plasma activity	Inflammatory	GPX3 protein associated with increased GPX3 mRNA levels in the kidney	[165]
ALS	No difference in plasma concentration; Lower GPX3 activity	-	GPX3 as a lead ALS risk gene, GPX3-*TNIP1* locus associated with ALS	[184,185,186]
ALS (Rats with mutant SOD1^H46R^)	Higher in serum in pre-symptomatic stage, decreased as disease progressed	-	-	[187]
Parkinson’s disease	Upregulated in blood	-	GPX3 as a potential biomarker for the disease	[192]
Cerebral thrombotic disorder patients	Decreased activity in blood	High ROS accumulation and rapid inactivation of nitric oxide	Exogenous GPX3 supplementation in plasma defends against thrombotic disorder	[195]
Kashin-Beck Disease	Reduced mRNA in chondrocytes	Elevated methylation of CpGs reduces antioxidant function and promotes chondrocyte apoptosis,	Reduced GPX3 accelerates KBD development	[206]
Hip fracture patients	Increased in bone samples	-	Increased GPX3 suggests increased antioxidative activity in bone samples.	[208]
Multiple organ failure and systemic inflammatory response patients	Decreased activity in serum	-	Early decrease of Se and GPX3 associated with multiple organ failure and systemic inflammatory response in ICU	[209]

**Table 3:** Summary table of the therapies based on GPX3, and potential disease biomarkers in disease progression.

Disease	Therapies Based on GPX3	Effect	Involved Pathways	Therapeutic Target	Potential Disease Biomarkers	Reference
AKI (IR induced)	GPX3-overexpression plasmid; vitamin Dvitamin D receptor; VDR agonist paricalcitol	Protects kidneys from oxidative stress injury.	Oxidative stress; Apoptosis	Maintaining renal GPX3 could be a strategy for AKI	Loss of renal GPX3 may promote renal oxidative stress injury	[50]
AKI (IR induced)	Apocynin; Neutrophil deficiency	Attenuated IR-induced renal functional impairment	Antioxidant	Enhancing GPX3 could be a strategic antioxidant defense against renal IR injury	-	[52]
AKI (Cisplatin induced)	Overexpression of GPX3 by virus injection	Prevents cisplatin induced AKI	Inhibiting oxidative stress and apoptosis of tubular cells	-	-	[41]
AKI (Vancomycin induced)	Recombinant Klotho	Alleviates vancomycin induced AKI	Upregulating anti-oxidative capacity via -JAK2/STAT3/GPX3 axis	-	-	[53]
AKI (Cardiac surgery associated)	-	-	-	-	GPX3 ratio as an early diagnostic marker for AKI	[54]
CKD	Overexpression of exogenous GPX3	Alleviates kidney fibrosis	Inhibits NADPH oxidase 2 and p38 mitogen activated protein kinase	-	-	[27]
CKD patients	High GPX3 activity with usual care	Sustained increase in renal function	Derepresses renal blood flow and a rise in eGFR	-	Inverse relationship between GPX3 activity and rate of eGFR decline	[59]
Atherosclerosis	-	-	-	-	GPX3 may impair endothelial function and result in atherosclerosis	[70,71]
MI (Mouse model of heart regeneration)	-	-	-	GPX3 as a target for heart regeneration therapy	-	[79]
Hypertension	-	-	-	-	GPX3 rs3828599-GG associated with hypertension incidence	[94]
Hypertensive patients with CKD	miR-338-3p	Target GPX3	-	GPX3 for managing hypertension and vascular damage in CKD	-	[95]
Aging	-	-	-	-	Decreased GPX3 activity may predict cardiovascular complications	[98]
AF	Mediterranean diet	Reduces vascular events rate	Modulates antioxidant activity of GPX3,	Nutritional strategy to prevent vascular events	-	[99]
Asthma	Two allelic mutations in GPX3 rs2070593	Prevents asthma development	-	GPX3 rs2070593 is as a protective locus for asthma	-	[115]
Hypersensitivity pneumonitis	-	-	-	GPX3 as a diagnostic biomarker with hypersensitivity pneumonitis	-	[129]
COVID-19	GPX3 rs8177412 variant genotype	Associated with severe COVID-19	-	GPX3 might be a complementing otheras a diagnostic tool for COVID-19 course prediction	-	[130]
Obesity	GPX3 rs922429	Protects against obesity classified by body fat percentage	-	-	-	[138]
T2DM	-	-	-	-	Lower GPX3 activity may predict carotid atherosclerosis in T2DM	[145,146]
Gestational DM	-	-	-	-	Low GPX3 activity associated with higher risk of gestational DM	[147]
Metabolic syndrome	Selenite	Enhances insulin receptor expression and adipocyte differentiation	Induces GPX3 expression; activates SP1	-	-	[26]
Metabolic syndrome	Overexpression of GPX3 in adipocytes	Suppresses pro-inflammatory gene and insulin resistance	Suppresses p47 and p67 subunits of NADPH-oxidase complex	-	-	[140]
IBD (sodium dextran sulfate induced)	Polyethylene glycol-modified Mo3Se4 nano flakes with multiple antioxidant enzymatic activities, including GPX3	Reduces disease activity index scores and reverses SS-induced IBD	Nrf2-keap1 antioxidant pathway	-	-	[166]
Hepatic IR injury	hiPSC-MSCs delivering GPX3; Recombinant GPX3	Ameliorates hepatic IR injury	Inhibits hepatic senescence and apoptosis; promotes liver regeneration	-	-	[171]
Hepatic IR injury	Paeoniflorin	Attenuates hepatic IR injury	Enhances hepatic GPX3 activity	Modulating micro-environment; Enhancing GPX3 supplementation might be a strategy for liver IR injury.	-	[172,173]
Hepatic injury (alcohol induced)	Up-regulation of GPX3	Protects against alcohol induced hepatic injury	-	-	-	[174]
ALS (Rats with mutant SOD1^H46R^)	-	-	-	Detects disease presence and stage progression	Potential serum biomarker for ALS	[187,188]
Cerebral thrombotic disorder	Exogenous GPX3 supplementation in plasma	Defends against thrombotic disorder	Restores nitric oxide-mediated platelet inhibition	-	Targeting GPX3 for thrombotic disorders	[195,201]
Kashin-Beck Disease	Selenium supplementation	Exerts protective effect	Reverses methylation status of GPX3, increases GPX3 expression, inhibits PI3K/Akt/c-fos pathway	-	-	[206]
Systemic inflammatory response syndrome	-	-	-	-	Decreased GPX3 activity as a predictor for this syndrome.	[209]
